# Reducing Effects of Whey Protein Hydrolysate on Coloration of Cured Sausages

**DOI:** 10.3390/foods13010013

**Published:** 2023-12-19

**Authors:** Shiro Takeda, Teppei Kanda, Abdulatef M. Ahhmed, Kazuki Sogawa, Keitarou Umezu, Masaya Ogata, Wataru Mizunoya, Ryoichi Sakata

**Affiliations:** 1 School of Veterinary Medicine, Azabu University, Sagamihara 252-5201, Japan; mizunoya@azabu-u.ac.jp (W.M.); sakata@azabu-u.ac.jp (R.S.); 2 Graduate School of Veterinary Science, Azabu University, Sagamihara 252-5201, Japan; teppei5613@gmail.com (T.K.); dejavu6160@gmail.com (K.U.); da2101@azabu-u.ac.jp (M.O.); 3 Center for Human and Animal Symbiosis Science, Azabu University, Sagamihara 252-5201, Japan; 4 Department of Nutritional Therapy, Graduate School of Medical Science, The Libyan Academy, Tripoli 79031, Libya; latef.ml@gmail.com; 5 School of Life and Environmental Science, Azabu University, Sagamihara 252-5201, Japan; sogawa@azabu-u.ac.jp

**Keywords:** nitrite, nitrosyl myoglobin, redness, reduction, sausage, whey protein hydrolysate

## Abstract

Curing produces a characteristic pink color during meat processing through the production of nitrosyl myoglobin (NOMb), which requires nitric oxide (NO). Nitrites and nitrates in coloring agents are crucial NO sources; however, a reducing agent is necessary to facilitate their chemical conversion to NO. This study aimed to investigate the effect of the reducing properties of whey protein hydrolysate (WPH) on the reddening of cured meat products. Cured and cooked sausage models were treated with WPH, which enhanced the reddening of the meat color and increased the a* value in the models compared with that of the controls. Additionally, ethanol-extracted WPH induced Fe^3^⁺ reduction, lowered oxidation–reduction potential, and decreased nitrite (NO_2_^−^) levels. Moreover, ethanol-extracted WPH promoted the formation of NOMb in myoglobin solution. This effect was also observed when ethanol-extracted WPH treated with maleimide was used, implying that certain peptides rather than the thiol group of WPH are involved in promoting NOMb formation. Furthermore, the peptides that decreased NO_2_^−^ levels were isolated from ethanol-extracted WPH, identified, and synthesized. These synthesized peptides, particularly the FFVAPFPEVFGK peptide, showed NO_2_^−^-reducing activity. Hence, WPH may promote the coloration of cured meat products through the reducing potential of the peptides contained within.

## 1. Introduction

The initial consumer impression of meat and meat products is predominantly influenced by coloration, making it one of the most intuitive factors influencing consumer purchasing decisions [1]. Curing agents—most notably nitrites (NO_2_^−^) and nitrates (NO_3_^−^)—are responsible for the characteristic pink color of meat products during meat processing. In addition, the fundamental function of NO_2_^−^ in the meat industry is to inhibit the growth of food-poisoning bacteria and to inhibit oxidation during storage [2]. The biochemical mechanisms underlying the development of the desirable pink coloration in cured meat products depend on the concentration of heme pigments and their redox states [3,4]. The pigment primarily responsible for imparting this pink color to cured meats is nitrosyl myoglobin (NOMb), which is a ferrous complex that forms between myoglobin and nitric oxide (NO) [5,6]. Hence, NO_2_^−^ and NO_3_^−^ also function as pivotal sources of NO during meat curing; however, they require the presence of a reducing agent—such as ascorbic acid—to facilitate their chemical conversion into NO [7,8]. Furthermore, the role of these reducing agents assumes significance in preventing residual NO_2_^−^ within the meat product since the persistence of NO_2_^−^ can form carcinogenic N-nitrosamines and engender toxicity within the final product [9].

The utilization of food by-products is garnering a lot of attention. Whey is a byproduct of cheese and casein production. Whey protein (WP) has gained increasing recognition as a value-added ingredient owing to its remarkably advantageous functional and nutritional attributes. WPs are widely used by dairies, bakeries, confectionaries, meat processing plants, canneries, and beverage manufacturers for their various functions in food quality and stability [10,11]. Furthermore, whey protein hydrolysate (WPH) is derived from WPs by enzymatic degradation, including various peptides, and finds utility in food processing. For example, WPH plays a role in pH stability and influences sensorial attributes such as color, flavor, and texture, although it can introduce a bitter taste during food processing [12]. There are several recent reports on the use of WPH in meat processing. WPH improves water retention, has antioxidant effects on proteins and lipids, inhibits protein aggregation, and has antibacterial effects on the freezing and thawing of meat products [13,14,15,16]. It exerts a favorable effect on the coloration of meat products during meat processing and promotes NOMb formation, which enhances the reddish color of cured meat products [17,18]. Furthermore, the peptides in acidic whey inhibit lipid oxidation and preserve meat color during the preservation of uncured and fermented meat products [19,20]. Since WPH exhibits antioxidant and/or reducing properties [21], it was hypothesized to impact the coloration of cured meat products; however, the detailed underlying mechanism is unknown.

This study aimed to examine the enhancement of reddish coloration in cured meat products by WPH with a particular focus on its reducing properties. We assessed the impact of adding WP or WPH on the coloration of a sausage model. Additionally, the ethanol (EtOH) extracts of WP and WPH were subjected to antioxidative and/or reducing capability evaluation tests and their capacity to decrease NO_2_^−^ levels. Finally, we aimed to identify the NO_2_^−^-decreasing peptides derived from among the tested WPHs. Our study findings suggest that WPH would be a useful ingredient in improving the quality of meat products.

## 2. Materials and Methods

### 2.1. Materials and Chemicals

Commercially obtained WP concentrate (Daiichirakuto EM-20; DAIICHI-KASEI Co., Ltd., Kyoto, Japan) and WPH (WPH; DAIICHI-KASEI Co., Ltd.) were used in all analyses performed in this study. In addition, fresh ground pork for the test was purchased from a local butcher shop (Sagamihara, Japan). They were edible grade and were purchased every time before the experiment.

EtOH, 2-amino-2-hydroxymethyl-1,3-propanediol (Tris), NaH_2_PO_4_, Na_2_HPO_4_, NaCl, NaNO_2_, sodium ascorbate, potassium ferricyanide, trichloroacetic acid, iron (II) chloride tetrahydrate, sulfanilamide, naphthyl ethylenediamine dihydrochloride, acetone, hydrochloric acid (HCl), acetic acid, sodium acetate trihydrate, and urea were purchased from FUJIFILM Wako Pure Chemical Corp. (Osaka, Japan), with a guaranteed reagent purity grade (Japanese Industrial Standards special grade) or maker-guaranteed reagent grade. In addition, sodium dodecyl sulfate (SDS) of molecular biology grade, 2-nitrobenzoic acid of SH group determination grade, CH_3_CN of high-performance liquid chromatography (HPLC) and LC/MS grade, and HCOOH of LC/MS grade were purchased from FUJIFILM Wako Pure Chemical Corp. Moreover, we used EDTA-2NA with over 99.5% purity (DOJINDO laboratories, Kumamoto, Japan), myoglobin (extracted from horse muscle: Biochemicals Reagent) with over 95% purity (Nacalai Tesque, Kyoto, Japan), Trolox with a purity of over 98% (Tokyo Kasei Kogyo Co., Ltd., Tokyo, Japan), and maleimide with a purity of 97% (Combi-Blocks Inc., San Diego, CA, USA). Also, trypsin of trypsin sequence grade was used for LC/MS (Roche Diagnostics GmbH, Mannheim, Germany).

### 2.2. WP and WPH EtOH Extracts

To prepare the WP and WPH EtOH extracts, 10% (weight/volume) of WP and WPH were suspended in distilled water. EtOH was added to the suspension to obtain a final EtOH concentration of 70% (volume/volume). The suspensions were kept overnight at 4 °C in the dark and centrifuged at 5000 rpm for 5 min at 4 °C. Subsequently, the supernatants were filtered through No. 5A filter paper (ADVANTEC, Tokyo, Japan), and each filtrate was dried in a rotary evaporator (N-1300; EYELA, Tokyo, Japan). The dried samples were suspended in distilled water, frozen at −80 °C, and lyophilized using a lyophilizer (FDU-1200; EYELA).

### 2.3. Sausage Model Preparation

To prepare the sausage model, 2% NaCl and 30 ppm NaNO_2_ were added to the ground pork and mixed on ice using a mortar. WP or WPH was added at 1.0, 2.5, or 5.0% weight per weight of the mixed ground pork and thoroughly mixed on ice using a mortar. For the positive control sample, 0.1% sodium ascorbate (*w*/*w*) was added, and the blank sample of ground pork was used with 2% NaCl and 30 ppm NaNO_2_. After mixing, the samples were immediately moved on ice and packed in a sanitary plastic bag. Then, they were heated at 75 °C for 20 min in a water bath (Thermominder SD; TAITEC, Koshigaya, Japan) to be prepared as each sausage model.

### 2.4. Meat Color Determination

The color of the sausage model was evaluated using a spectrophotometer (MINOLTA CM-3500; Konica Minolta Sensing, Inc., Tokyo, Japan) set up with a D65 light source, reflectance rejection, 10° field of view, and reflectance measurement. The results are indicated as lightness (L*), redness (a*-redness/greenness), and yellow (b*-yellowness/blueness) values. The measurements were conducted in triplicate as a technical replication and in three to four independently prepared samples for biological replication.

### 2.5. Fe^3^⁺-Reducing Activity

The Fe^3^⁺ reduction reaction was measured using a method described by Ferreira et al. [22] with modifications. The sample (100 µL) was suspended in distilled water with 100 µL of 1% (*w*/*v*) potassium ferricyanide and 100 µL of 0.2 mol/L phosphate buffer (pH 6.6). The solution was heated at 50 °C for 20 min. After cooling on ice, 100 µL of 10% (*v*/*v*) trichloroacetic acid solution was added and mixed. Subsequently, 100 µL of the mixed solution, 100 µL of distilled water, and 20 µL of 0.1% iron (Ⅱ) chloride tetrahydrate solution were added to a 96-well plate and allowed to stand for 10 min in the dark at approximately 25 °C. Thereafter, the absorbance was measured at 700 nm using an UV-1800 spectrophotometer (Shimadzu, Kyoto, Japan). The reducing capacity was calculated as the Trolox equivalent per 1 mg of extract (mg TE/mg) using a calibration curve prepared with Trolox. The measurements were conducted on three independently prepared samples for replication.

### 2.6. Oxidation–Reduction Potential Measurement

WP and WPH EtOH extracts were suspended in distilled water, and the respective oxidation–reduction potential (ORP) values were measured using an ORP sensor (OR-101S; Kasahara Chemical Instruments Corp., Saitama, Japan) equipped with a KP-10F pH/ORP meter (Kasahara Chemical Instruments Corp.). The measurements were conducted on four independently prepared samples for replication.

### 2.7. NO_2_^−^-Reducing Activity

Each sample was incubated in a sodium nitrite solution, in which the residual NO_2_^−^ in the solution was measured, and the NO_2_^−^-decrease rate was calculated. Briefly, 50 µL of the sample solution was added to 1.0 mL of 1 ppm sodium nitrite diluted in 0.1 mol/L acetate buffer (pH 5.5) in a test tube. Nitrogen gas was blown into the prepared solution for 15 min and the headspace for 5 min. Afterward, the mixed sample solutions were sealed and kept in the dark for 1 h at approximately 25 °C.

Residual NO_2_^−^ levels were determined according to a previous method [23]. Briefly, 0.5% sulfanilamide solution and 0.2% naphthyl ethylenediamine dihydrochloride solution were added to the tested sample solution at 40% (*v*/*v*) and incubated for 20 min. After incubation, the absorbance was measured at 540 nm using a UV-1800 spectrophotometer (Shimadzu) or a Multiskan FC microplate reader (Thermo Fisher Scientific, MA, USA). The residual NO_2_^−^ levels were estimated from a calibration curve prepared using a sodium nitrite solution, and the decreased rate from the initial sodium nitrite concentration was determined as the NO_2_^−^-reducing activity. For measurements of NO_2_^−^-reducing activity of WP and WPH EtOH extracts, the test was conducted on five independently prepared samples for replication. In addition, it was conducted in triplicates for the fractionated samples with gel filtration and HPLC.

### 2.8. Nitrosyl Myoglobin-Forming Activity

The total heme pigment concentration was determined based on a 75% acetone–0.7% hydrochloric acid extraction method with modifications [17,24,25,26]. Briefly, 0.4 mL of 0.625% myoglobin (Mb) solution, 1.9 mL of 0.1 mol/L acetate buffer (pH 5.5), 7.5 mL of acetone, and 0.2 mL of hydrochloric acid were mixed and allowed to react for 60 min on ice in the dark. The solution was filtered through filter paper (No. 6; ADVANTEC TOYO, Tokyo, Japan) and collected in a brown test tube. The absorption spectra of the sample solutions were determined using a spectrophotometer (UV-1800; Shimadzu, Kyoto, Japan) at 340–720 nm. The absorbance at 383 nm was used to measure the total heme dye content.

The nitrosyl heme dye level was determined using the modified 75% acetone extraction method [17,24,25,26]. Briefly, 0.4 mL of 0.625% Mb solution was added to 2.0 mL of the test sample. Nitrogen gas was bubbled on ice for 15 min in the solution and 5 min in the headspace of the tube. Thereafter, the tube was sealed, and 0.1 mL of 0.25% NaNO_2_ solution was anaerobically added to the sample solution. The sample solution was subsequently incubated in a water bath at 75 °C. Acetone (7.5 mL) was added to the heated sample solution, which was kept on ice in the dark for 30 min before filtration through No. 6 filter paper (ADVANTEC). The absorption spectrum of the filtrate was measured at 340–720 nm using a spectrophotometer (UV-1800; Shimadzu), and the absorbance at 395 nm was used to measure the amount of nitrosyl heme dye. The absorbances of the total and nitrosyl heme dyes were measured at 383 and 395 nm, respectively. These values were used to determine the NOMb forming rate using the following formula:

NOMb forming rate (%) = (absorbance at 395 nm × 1.2)/absorbance at 383 nm × 100

The measurements were conducted on three independently prepared samples for replication.

### 2.9. Maleimide Modification of WPH

Lyophilized WPH EtOH extract was dissolved in 0.1 mol/L of Tris-HCl (pH 7.0), after which 77.8 mL of 1.0 mol/L maleimide solution was added to 700 mL of 10 mg/mL WPH solution and incubated for 1 h at approximately 25 °C with mixing. The solution was kept at −80 °C overnight and lyophilized. The lyophilized powder was used as the maleimide-treated WPH (thiol group blocked WPH). Owing to the presence of maleimide, the percentage of WPH weight was modified to 48% in the final maleimide-treated WPH powder.

### 2.10. Measurement of Thiol Group Concentration

The thiol group concentration was determined using a previously reported method [27]. Briefly, a 0.4% 2-nitrobenzoic acid solution was prepared by adding 2-nitrobenzoic acid to 50 mmol/L Tris-HCl (pH 6.8) buffer with 2% SDS, 48% urea, and 0.292% EDTA. The sample was suspended in distilled water, and 0.5 mL of the suspension, 2.5 mL of Tris-HCl buffer (50 mmol/L, pH 6.8), and 20 µL of 0.4% 2-nitro-benzoic acid solution were thoroughly mixed and kept at approximately 25 °C in the dark for 1 h. Thereafter, the mixture was centrifuged at 5000 rpm for 7 min, and the absorbance of the yielded supernatant was determined at 412 nm using a spectrophotometer (UV-1800, Shimadzu). The thiol group concentration was estimated using the formula:

Thiol group (mmol/g) = (73.53 × absorbance at 412 nm)/sample weight

The measurements were conducted on three independently prepared samples for replication.

### 2.11. Isolation and Identification of NO_2_^−^-Reducing Peptides

To isolate and identify the NO_2_^−^-reducing peptides from the lyophilized WPH EtOH extract, gel filtration chromatography was performed using Sephadex G-25^®®^ superfine gel (GE Healthcare, Uppsala, Sweden), which was equilibrated using 0.01 N HCl in a 4.5 × 53-cm column. The elution was performed using 0.01 N HCl as the eluent at a flow rate of 4.0 mL min^−1^ at approximately 25 °C. The fractions were collected from 75 to 245 min every 1 min using a Bio-collector AC-5750 (ATTO, Tokyo, Japan). All fractions were lyophilized, dissolved in 1.0 mL distilled water, and stored at −80 °C until the NO_2_^−^-reducing assay was performed. Additionally, the active fraction obtained by gel filtration chromatography was re-fractionated using a JASCO LC−1500 intelligent HPLC system (JASCO, Hachioji, Japan). The fractionation conditions were the same as those described in a previous study [28]. The fractionated samples were lyophilized, dissolved in 1.0 mL distilled water, and stored at −80 °C until the NO_2_^−^-reducing assay was performed. The active fractions were freeze-dried and used for subsequent analyses.

The amino acid sequences of the peptides in the active fractions were identified using a previously reported protocol [29]. The lyophilized samples were rehydrated for 45 min in 10–30 μL of 25 mmol/L Tris-HCl/20% CH_3_CN containing 25 ng/L trypsin. Following the removal of the unabsorbed solution, the rehydrated samples were incubated in 10–20 μL buffer containing 50 mmol/L Tris-HCl/20% CH_3_CN for 20 h at 37 °C. The peptide fragments were injected into a 0.3 × 5 mm L-trap column (Chemicals Evaluation and Research Institute, Saitama, Japan) and a 0.1 × 50 mm Monolith analytical column (AMR, Tokyo, Japan) attached to an HPLC system (Nanospace SI-2; Shiseido Fine Chemicals, Tokyo, Japan). The flow rate of the mobile phase (solvent A and B) was 1 μL min^−1^. Solvent A was 2% *v*/*v* CH_3_CN and 0.1% *v*/*v* HCOOH, and solvent B was 90% *v*/*v* CH_3_CN and 0.1% *v*/*v* HCOOH; the composition of the mobile phase was changed every 35 min. The gradient system for the mobile phase was as follows: 5–50% solvent B for 20 min; 50–95% solvent B for 1 min; 95% solvent B for 3 min; 95–5% solvent B for 1 min; and 5% solvent B for 10 min. The purified peptides were introduced into an LTQ-XL ion trap mass spectrometer (Thermo Scientific, San Jose, CA, USA) using an attached pico tip (New Objective, Woburn, MA, USA). Mass spectra (MS) and tandem mass spectra (MS/MS) of the peptides were obtained in a data-dependent manner. The MASCOT search engine (Matrix Science, London, UK) was used to identify proteins from the MS and MS/MS data of the peptides. Mass data of the peptides were matched by searching the NCBI database using the MASCOT engine. The *Bos taurus* database was used in this study. The minimum significance threshold level for the probability-based MASCOT/MOWSE score was set to 5% [30]. Finally, the identified peptides were synthesized by Scrum Inc. (Tokyo, Japan) and purified to 98% purity using an HPLC column.

### 2.12. Statistical Analysis

Student’s *t*-test was used to analyze the L*, a*, and b* values in the sausage model, antioxidative and reducing activities of WP and WPH EtOH extracts, NOMb formation ratio in Mb solution by WP and WPH EtOH extracts, and thiol group levels in WPH EtOH and maleimide-treated WPH EtOH extracts. A *p*-value of less than 0.05 (*p* < 0.05) was considered statistically significant.

## 3. Results

### 3.1. Meat Color of the Sausage Model with WP and WPH

**Figure 1:**
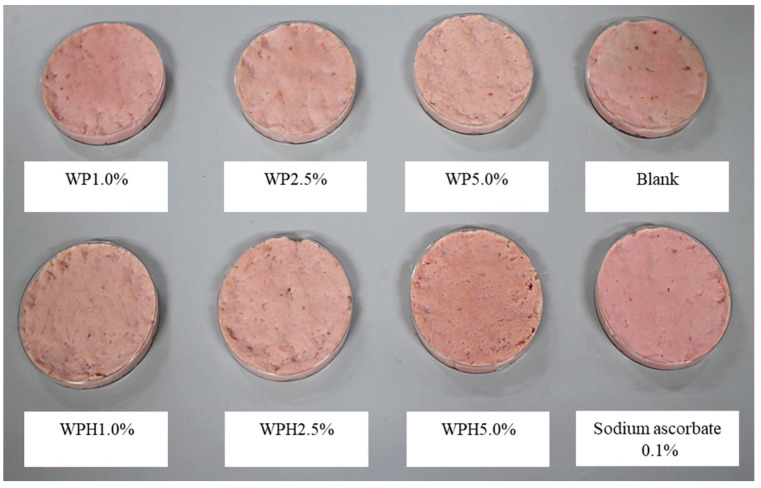
Visual color of the sausage model treated with whey protein (WP), whey protein hydrolysate (WPH), or sodium ascorbate. WP, WPH, and sodium ascorbate were added to ground pork at weight per weight.

As shown in Figure 1, the meat color of the sausage model was affected by WP or WPH addition. The sausage model treated with 5.0% WPH appeared to be the most reddish among the tested sausages. Similarly, the sausage model supplemented with 1.0% WP also showed a reddish appearance compared to the control; however, this reddening effect was not observed at higher WP concentrations.

No significant differences in L*, a*, and b* values were found among all the tested sausages using one-way ANOVA (Table 1). However, the a* value of sausages treated with WPH tended to be higher than that of the control and sausages treated with WP.

In particular, the a* value of 5.0% WPH-treated sausage was significantly higher than that of 5.0% WP-treated sausage (*p* < 0.05). Meanwhile, the L* value of 5.0% WPH-treated sausage was significantly lower than that of 5.0% WP-treated sausage (*p* < 0.05).

### 3.2. Antioxidant and Reducing Activities of WP and WPH

The WP and WPH EtOH extracts were subjected to Fe^3^⁺-reducing activity assays (Table 2). This examination is widely used to assay for antioxidant activity. The reduction reactions of Fe^3^⁺ to Fe^2^⁺ in the WP and WPH EtOH extracts were assayed. The Fe^3^⁺-reducing activity of the WPH EtOH extract was significantly higher than that of the WP EtOH extract at 25 and 50 mg/mL contents (*p* < 0.05). In addition, all ORP values of the WPH EtOH extract were significantly lower than those of the WP EtOH extract at the same contents (*p* < 0.05). Moreover, the NO_2_^−^-decreasing ratio of the WP and WPH EtOH extracts was measured. The reduction of NO_2_^−^ ions results in the release of NO, which is suggested to lead to a decrease in NO_2_^−^ concentration. The NO_2_^−^-decreasing ratio of the WPH EtOH extract was significantly higher at all levels than that of the WP EtOH extract (*p* < 0.05).

### 3.3. NOMb-Forming Activity of WP and WPH

**Figure 2:**
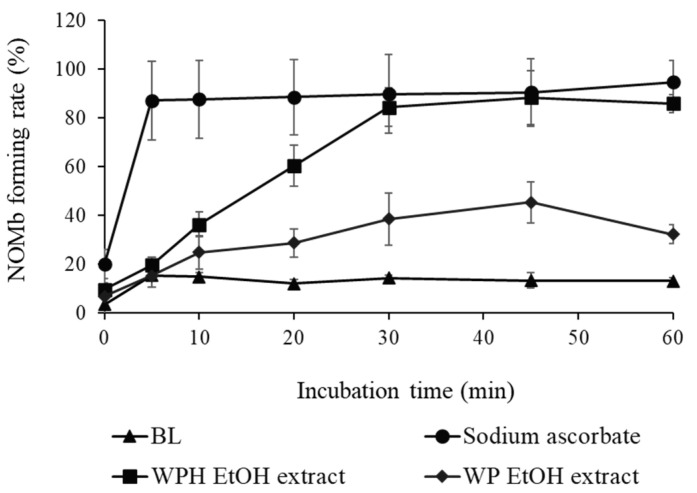
Nitrosyl myoglobin (NOMb)-forming activity of WP EtOH and WPH EtOH in the myoglobin (Mb) solution. The change in NOMb-forming rate of WP EtOH and WPH EtOH with incubation time is shown. Error bars represent SD. Data are presented as mean ± SD (*n* = 3). BL, blank.

**Figure 3:**
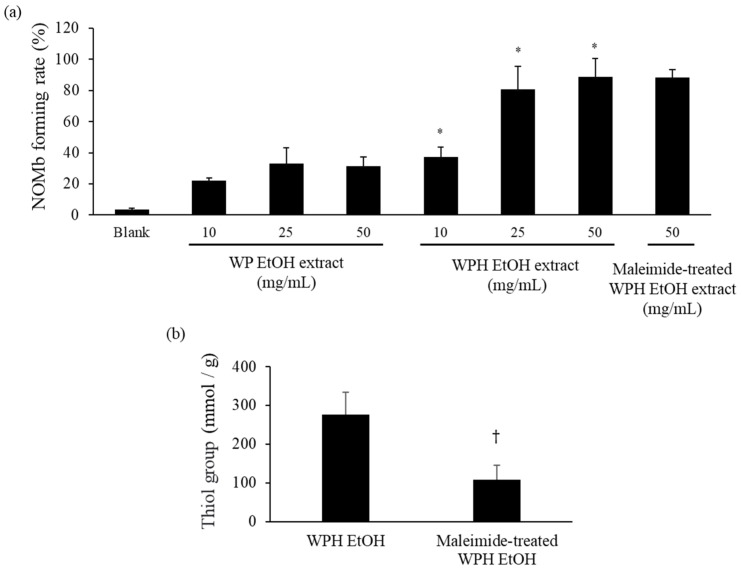
NOMb-forming activity of WP EtOH, WPH EtOH, and maleimide-treated WPH EtOH in the Mb solution. (**a**) NOMb forming rate 30 min after heating. (**b**) Thiol group levels in the maleimide-treated WPH. Error bars represent SD. Data are presented as mean ± SD (*n* = 3). * indicates a significant difference between WP EtOH and WPH EtOH at the same level using Student’s *t*-test (*p* < 0.05). † indicates a significant difference relative to WPH EtOH (*p* < 0.05).

As shown in Figure 2, the NOMb-forming rate in the sodium ascorbate group, as the positive control, was remarkably elevated after incubation at 75 °C, and that of the blank—the negative control—was scarcely observed in the forming of NOMb. Although inferior to the sodium ascorbate group, the WPH group showed a gradually increasing rate of NOMb formation until 30 min after heating, after which the NOMb formation rate remained constant. The WP group also showed an increase in the NOMb formation rate after incubation, which was clearly lower than those of the WPH and sodium ascorbate groups. The NOMb-forming ratio at 30 min after heating showed that all WPH levels yielded significantly higher values than WP at the same content (*p* < 0.05; Figure 3a). In addition, 50 mg/mL maleimide-treated WPH demonstrated approximately the same NOMb-forming ratio as 50 mg/mL WPH. The thiol group level of maleimide-treated WPH was 107.84 mmol/g, which was significantly lower than that of WPH at 276.96 mmol/g (*p* < 0.05; Figure 3b).

### 3.4. Isolation and Identification of NO_2_^−^-Decreasing Peptides from WPH

**Figure 4:**
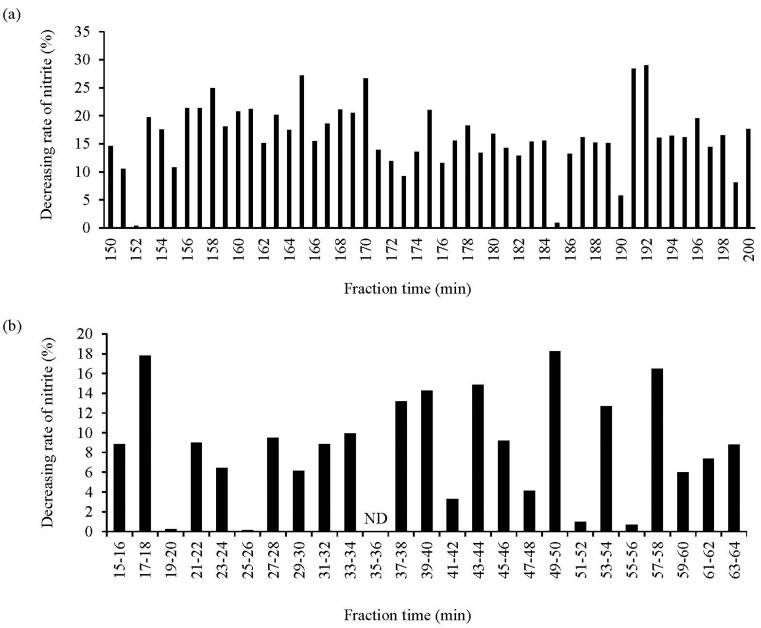
Assessment of the NO_2_^−^-decreasing activity of different WPH EtOH fractions. (**a**,**b**) show the NO_2_^−^-decreasing rate of the fractions based on gel filtration chromatography (during a 150–200 min fractionation time) and reversed-phase chromatography (during a 15–64 min fractionation time), respectively. The other fractions not shown in the figure had low activity. Data are expressed as the mean of triplicate experiments. ND, not detected.

The potent NO_2_^−^-decreasing peptides derived from the WPH EtOH extract were investigated. The WPH EtOH extract was subjected to gel filtration chromatography using a Sephadex G-25 column, and the NO_2_^−^-decreasing rate of each fraction was assayed. The results suggested that the fraction eluted after 192 and 191 min of sample loading exhibited the highest and next highest NO_2_^−^-decreasing ratios, respectively, among all fractions (Figure 4a). The two fractions were subjected to subsequent chromatography since they demonstrated high NO_2_^−^-decreasing ratios close to the retention time. Moreover, the eluted fractions were mixed and subjected to reversed-phase chromatography using HPLC, and the NO_2_^−^-decreasing ratios of the 25 fractions in the HPLC chromatogram were assessed; the fractions eluted at retention times of 17–18 (FR1), 49–50 (FR2), and 57–58 (FR3) min exhibited greater NO_2_^−^-decreasing ratios (Figure 4b). Also, FR2 had the highest NO_2_^−^-decreasing ratio among all fractions.

To identify the source of NO_2_^−^-decreasing activity, FR1, FR2, and FR3 were subjected to LC-MS/MS analysis. Two peptides in FR1, FR2, and FR3 were detected and identified, from which the source proteins were determined (Table 3). The peptides HIQKEDVPSER, FFVAPFPEVFGK, and EVLENLLR in FR1, FR2, and FR3, respectively, were suggested to be derived from α-casein S1. In addition, KEAVALK in FR1 and TPEVDDEALEKFDKALK in FR2 and FR3 were derived from the Tudor domain containing 15 proteins and β-lactoglobulin, respectively. Moreover, all the identified peptides were synthesized, and their NO_2_^−^-decreasing activities were assessed. Among the synthesized peptides, the NO_2_^−^-decreasing ratios of FFVAPFPEVFGK in FR2 and EVLENLLR in FR3 were 60.7 and 52.3%, respectively. Furthermore, these reduction ratios corresponded to 0.17 and 0.16 mM of sodium ascorbate equivalent relative to the NO_2_^−^-decreasing activity of sodium ascorbate.

## 4. Discussion

WPH is now recognized as a value-added ingredient because of its highly functional benefits in food processing [12]. This study focused on the reducing effect of WPH and investigated the mechanism by which WPH promotes NO_2_^−^-induced red coloration during meat processing.

Except for the positive control which was treated with 0.1% sodium ascorbate, the 5% WPH enhanced the visual redness of the sausage model compared with that of the other sausages (Figure 1). In addition, the a* value of the 5% WPH-treated sausage model was significantly higher than that of the 5% WP-treated sausage (Table 1). Furthermore, the a* values of the sausage model tended to be higher at the other concentrations, although no significant differences were observed. WPH includes various peptides derived from WP. Thus, treatment of cured meat products with WPH was suggested to promote meat reddening, indicating that the peptides in WPH contributed to the development of meat redness. A previous study concluded that adding WPH to cured meat products increased the redness of the products, consistent with the present study [17,18]. In those previous studies, WPH was examined as a color formation accelerator in meat products such as hams and sausages, and the extent to which it stabilizes heme pigments was investigated [17,18]. The main pigment in cured and cooked meat products is nitrosyl hemochromogen [31,32]; however, NO_2_^−^ is reduced and converted into NO during the curing process before cooking and binds to Mb, initiating the reaction involved in NOMb formation [7]. Thus, the present study focused on the reducing capacity of WPH and its role in enhancing meat product coloration.

Previous studies have reported the reducing and antioxidant potentials of WP peptides, and WPH has also been used in the food industry [12,33,34]. In the current study, the WPH EtOH extract showed Fe^3^⁺ reduction and promoted electron supply to Fe^3^⁺ (Table 2). In addition, the WPH EtOH extract decreased the ORP values, indicating its excellent reducing power. Moreover, the WPH EtOH extract lowered NO_2_^−^ levels, that could inhibit NO_2_^−^ and oxygen interactions as well as its chemical reactivity. Thus, the peptides in the tested WPH were suggested to have potential reducing activity. Tinbergen [35] inferred that the low-molecular weight active fraction from meat (mainly its SH group) could be involved in nitrite reduction. Thus, the peptides in WPH are thought to play a role in the formation of NOMb during meat processing through NO_2_^−^ reduction, potentially contributing to enhancing red coloration.

Myoglobin is the principal protein influencing meat color and is related to the development of red coloration through NO_2_^−^ in meat processing. WPH formed NOMb from Mb more rapidly than WP in the Mb solution but not as rapidly as sodium ascorbate (Figure 2 and Figure 3a), which is consistent with WPH’s ability to promote reddening of the tested cured meat (Figure 1 and Table 1). Since the thiol group is a well-established reducing agent, the thiol group in WPH potentially contributed to promoting the reddening of the tested cured meat. According to a previous study, the thiol group of low molecular fractions is believed to stimulate the formation of NOMb in meat processing [35]. Additionally, thiol amino acids derived from acid whey assist in preserving pink coloration during the storage of meat products [19]. In the present study, we investigated the NOMb formation reaction of WPH after modification with thiol groups by maleimide treatment, which reduced the thiol concentration to approximately one-third (Figure 3b). The results indicated that maleimide-treated WPH continued to be effective in elevating NOMb formation (Figure 3a). Hence, the thiol group is thought to have had a minor impact on the reduction reaction of WPH associated with NOMb formation. Hence, peptides with inherent reducing properties may have played a more important role.

We focused on investigating potential peptides derived from WPH with reducing effects, especially NO_2_^−^-decreasing effects. The fractionation and NO_2_^−^-reducing assay identified six NO_2_^−^-decreasing peptides from WPH that contributed to NOMb formation. The peptides were suggested to be derived from α-casein S1, Tudor domain protein, and β-lactoglobulin. In general, casein is not a major constituent of whey. However, as whey is a byproduct of cheese production and casein protein preparation, cheese whey may contain casein degradation products from rennet and lactic acid bacteria starter [36]. Although the detailed manufacturing process of the WPH used in the current study is unknown, the tested WPH constituents were potentially derived from cheese whey. Thus, the peptides derived from casein might have been derived from cheese whey.

Vavrusova et al. [21] indicated antioxidant peptide candidates from enzymatically hydrolyzed WP. However, the antioxidant peptides detected in the present study did not have the same amino acid sequences as those of the reported peptides (Table 3). L-Lysine/L-arginine/L-cysteine amino acids promote color development by NaNO_2_ in meat processing; color development would result from the antioxidation of these amino acids [37]. As shown in Table 3, lysine and arginine are present in the amino acid sequences of all the listed peptides. Thus, the NO_2_^−^-decreasing effect of these peptides may be owing to the presence of lysine and arginine in each peptide. Moreover, cysteine associated with a thiol group was not observed. Hence, as described above, the thiol group had a minor impact on the reducing effect of WPH and the promotion of NOMb formation in the present study. According to the BIOPEP database, the peptide HIQKEDVPSER in FR1 is considered an antioxidant peptide, and FFVAPFPEVFGK in FR2 is a known angiotensin-converting enzyme (ACE) inhibitor and anti-cancer peptide [38]. The HIQKEDVPSER peptide has antioxidant properties, and it can be inferred that this ability contributed to the NO_2_^−^-reducing effect. In addition, FFVAPFPEVFGK was suggested to have not only ACE inhibition and anti-cancer activity but also reducing and/or antioxidant and NO_2_^−^-reducing activity.

WPH seems to express bitterness during food processing [12]. It also inhibits lipid oxidation and improves product texture [39,40]. The effects of WPH on the palatability of the tested sausage model were not evaluated in this study. Further studies are needed to investigate the several organoleptic properties of cured meat products treated with WPH.

## 5. Conclusions

In conclusion, this study demonstrated that WPH promotes the red coloration of the sausage model, attributed to the reducing properties of WPH, which contributes to the promotion of NOMb formation. In addition, the reducing effects of WPH peptides contribute to NOMb formation in Mb solution to a greater extent rather than the effect of thiol group-containing amino acids in WPH. Moreover, the NO_2_^−^-reducing peptides, including the FFVAPFPEVFGK peptide, were identified in the WPH used in this study. Therefore, we believe that whey peptides are a useful ingredient to enhance meat functional properties and the quality of meat products.

## Figures and Tables

**Table 1:** Measurement of L*, a*, and b* values in the sausage model.

	Concentration (% *w*/*w*)	L*	a*	b*
Blank	—	63.24 ± 5.66	5.72 ± 0.51	8.61 ± 1.38
Sodium ascorbate	0.1	61.25 ± 1.63	7.76 ± 1.69	7.81 ± 0.94
WP	1.0	55.34 ± 0.62	6.04 ± 0.99	8.11 ± 1.17
	2.5	54.45 ± 3.75	5.80 ± 1.25	7.93 ± 0.80
	5.0	68.46 ± 0.84	5.82 ± 0.21	10.03 ± 0.58
WPH	1.0	56.56 ± 0.82	6.80 ± 1.04	9.46 ± 1.88
	2.5	57.24 ± 3.33	6.79 ± 2.19	8.72 ± 0.04
	5.0	64.47 ± 1.49 ^†^	6.83 ± 0.39 ^†^	9.20 ± 0.17

^†^ *p* < 0.05 vs. the value of WP at the same concentration. WP, whey protein; WPH, whey protein hydrolysate. Data are presented as mean ± standard deviation (SD) (*n* = 3–4).

**Table 2:** Antioxidative and reducing activities of WP and WPH EtOH extracts.

	WP EtOH Extract			WPH EtOH Extract		
Concentration (mg/mL)	10	25	50	10	25	50
Fe^3+^ reduction Trolox equivalent value (µmol/L)	11.24 ± 8.74	17.56 ± 12.32	31.25 ± 25.35	27.79 ± 11.98	63.05 ± 11.64 *	104.87 ± 12.32 *
ORP value (mV)	228.0 ± 15.7	210.0 ± 5.6	206.5 ± 13.9	181.3 ± 5.7 *	168.5 ± 9.3 *	160.0 ± 15.9 *
NO_2_^−^-decreasing ratio (%)	29.86 ± 5.80	27.77 ± 7.08	22.62 ± 6.29	42.08 ± 6.89 *	40.82 ± 2.03 *	33.95 ± 5.00 *

* *p* < 0.05 vs. the value of WP-EtOH at the same concentration. EtOH, ethanol; ORP, oxidation–reduction potential. Data are presented as mean ± SD (*n* = 3–5).

**Table 3:** Identification of NO_2_^−^-decreasing peptides from the WPH extract.

Fraction	Amino Acid Sequence	Origin of Protein Fragment ^※^	NO_2_^−^-Decreasing Activity (%) ^†^
FR1	HIQKEDVPSER	α-casein S1	33.7
	KEAVALK	Tudor domain containing 15 proteins	40.48
FR2	FFVAPFPEVFGK	α-casein S1	60.7
	TPEVDDEALEKFDKALK	β-lactoglobulin	23.5
FR3	TPEVDDEALEKFDKALK	β-lactoglobulin	23.5
	EVLENLLR	α-casein S1	52.3

FR1, FR2, and FR3 were obtained from the fractions eluted at retention times of 17–18, 49–50, and 57–58 min in reversed-phase chromatography, respectively. ^※^ The *Bos taurus* database was used for the protein search. ^†^ The NO_2_^−^-decreasing activity of the synthesized peptide was assayed.

## Data Availability

All data are contained within the manuscript.
